# Exploration of the core genes in ulcerative interstitial cystitis/bladder pain syndrome

**DOI:** 10.1590/S1677-5538.IBJU.2020.1104

**Published:** 2021-02-28

**Authors:** Hao Wu, Quan-Xin Su, Zi-Yi Zhang, Ze Zhang, Sheng-Lin Gao, Chao Lu, Li Zuo, Li-Feng Zhang

**Affiliations:** 1 People's Hospital of Nanjing Medical University Department of Urology Changzhou China Department of Urology, Affiliated Changzhou No. 2 People's Hospital of Nanjing Medical University, Changzhou, China;; 2 Dalian Medical University Dalian China Dalian Medical University, Dalian, China

**Keywords:** Cystitis, Interstitial, Computational Biology, Genes

## Abstract

**Objective:**

Interstitial cystitis (IC)/bladder pain syndrome (BPS) is a chronic inflammatory disease that can cause bladder pain and accompanying symptoms, such as long-term urinary frequency and urgency. IC/BPS can be ulcerative or non-ulcerative. The aim of this study was to explore the core genes involved in the pathogenesis of ulcerative IC, and thus the potential biomarkers for clinical treatment.

**Materials and Methods:**

First, the gene expression dataset GSE11783 was downloaded using the Gene Expression Omnibus (GEO) database and analyzed using the limma package in R to identify differentially expressed genes (DEGs). Then, the Database for Annotation, Visualization and Integrated Discovery (DAVID) was used for Gene Ontology (GO) functional analysis, and the Kyoto Encyclopedia of Genes and Genomes (KEGG) was used for pathway enrichment analysis. Finally, the protein-protein interaction (PPI) network was constructed, and key modules and hub genes were determined using the STRING and Cytoscape software. The resulting key modules were then analyzed for tissue-specific gene expression using BioGPS.

**Results:**

A total of 216 up-regulated DEGs and 267 down-regulated genes were identified, and three key modules and nine hub genes were obtained.

**Conclusion:**

The core genes (CXCL8, CXCL1, IL6) obtained in this study may be potential biomarkers of interstitial cystitis with guiding significance for clinical treatment.

## INTRODUCTION

Interstitial cystitis (IC)/bladder pain syndrome (BPS) is a chronic inflammatory disease characterized by chronic bladder pain and accompanying symptoms, such as frequent urination and urgency (1). The European Society for the Study of Interstitial Cystitis (ESSIC) defines its diagnosis based on chronic bladder pain and discomfort lasting for six months or longer, with at least one accompanying urinary tract symptom, such as frequent or urgent urination. However, it is necessary to rule out diseases with easily confusable symptoms, and to deal with other organ symptoms, as well as cognitive, behavioral, emotional and sexual factors (2). Female patients account for the majority of patients, the prevalence among women is between 2.7% and 6.5%, and the male-to-female prevalence ratio is roughly 5:1 or higher (1, 3). Subtypes include ulcerative and non-ulcerative IC/BPS. Ulcerative IC/BPS is defined by the presence of one or more different inflammatory lesions in the bladder. Some studies have indicated that there may be differences in the mechanism, performance, and symptoms between the two subtypes (4–6). At present, most oral drugs for this disease have no apparent effect, and the main goal of treatment is therefore to relieve symptoms (7). Therefore, understanding the pathogenesis and gene expression related to IC/BPS will aid in the development of better clinical strategies.

In this study, the GSE11783 dataset was downloaded from the Gene Expression Omnibus (GEO) database, and R was used for analysis to obtain differentially expressed genes (DEGs). Subsequently, a series of analyses were conducted, including Gene Ontology (GO) function analysis, Kyoto Encyclopedia of Genes and Genomes (KEGG) pathway enrichment analysis, followed by the construction of protein-protein interaction (PPI) networks. The results of this study could have a positive impact on IC research and provide new ideas for clinical diagnosis and treatment.

## MATERIALS AND METHODS

### Microarray Data

The microarray expression profiling dataset GSE11783, which was based on the GPL570 platform and saved by Gamper M et al., (8) was downloaded from the GEO database. This profile contained five IC patients and six normal control patients. From each IC patient, two bladder biopsies were analyzed, one from an ulcer area, and one from a non-ulcer area. Basic factors such as the patient's age, gender, and health condition are unknown. These data are available for free via the Internet.

### Data Preprocessing and Identification of DEGs

The GSE11783 original data set and GPL570 platform annotation file were downloaded. The raw expression data were preprocessed using the RMA algorithm from the affy package in R, and the limma package was used to perform difference analysis, identify the DEGs between ulcerative IC group, non-ulcerative IC group and normal control group, and to calculate the adjusted P value and long FC. After adjustment, p <0.05 and |log FC| >2, so the conditions for DEGs were met. Volcano plots were generated using the ggplot2 package in R, and heat maps of the top 50 up-regulated genes and top 50 down-regulated genes were generated using Heml software.

### GO functional analysis and KEGG Pathway enrichment analysis of DEGs

GO analysis, which can predict protein function, is divided into three categories: cellular component (CC), biological process (BP), and molecular function (MF). KEGG is a database that integrates chemical, genomic, and systemic functional information. The DAVID tool can provide a functional interpretation of gene lists obtained from genomic studies. GO functional analysis and KEGG pathway enrichment analysis of up- and down-regulated DEGs were performed using DAVID tools. Gene counts ≥5 and p <0.05 were used as screening conditions.

### Construction of PPI Network and Determination of Key Modules

The PPI network was constructed using STRING, an online database that can predict protein interactions. Previously obtained DEGs were mapped to this database, and an interaction score >0.4 was considered statistically significant. Cytoscape, a software that can visualize intermolecular interaction networks, was used to visualize the PPI network. Key modules in the PPI network were identified using MCODE, and the criteria selected were MCODE scores >5, max depth=100, degree cut-of=2, node score cut-off=0.2, and k-score=2. These modules were then subjected to GO functional analysis and KEGG pathway enrichment analysis using DAVID.

### Tissue-specific expression analysis of key module

A tissue-specific expression analysis of genes from the key modules was performed using the online tool BioGPS. The criteria for being highly tissue-specific were: [1] the tissue-specific expression level was over ten times greater than the median, and [2] the highest expression level was over three times greater than the second highest expression level (9).

### Identification of hub genes

The cytoHubba plugin was used to determine the hub genes for this PPI network. Four methods (Degree, Radiality centrality, Closeness centrality, EPC) were used to assess hub genes (10).

## RESULTS

### Identification of DEGs

**Figure 1:**
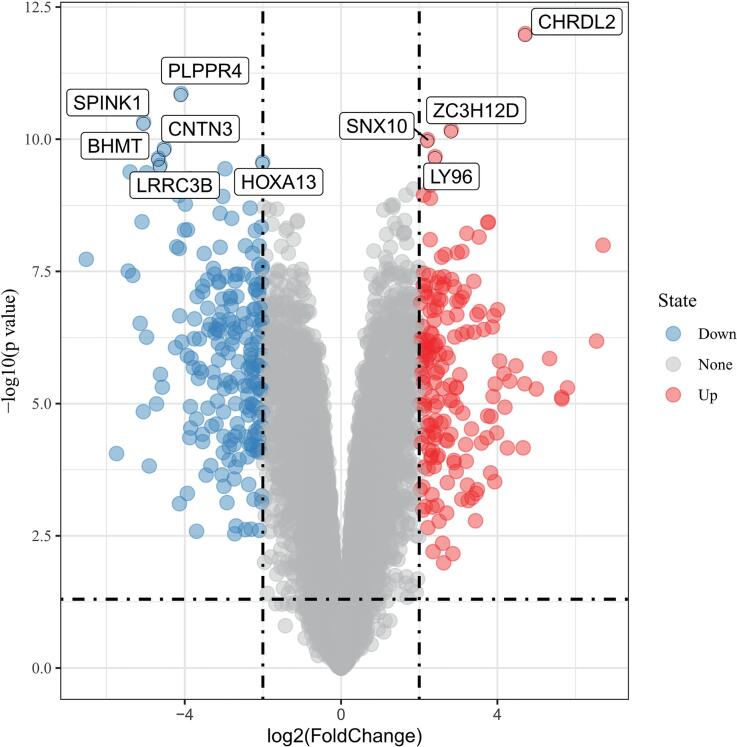
The volcano plots of differentially expressed genes (DEGs). Red represents up-regulated DEGs and blue represents down-regulated DEGs.

**Figure 2:**
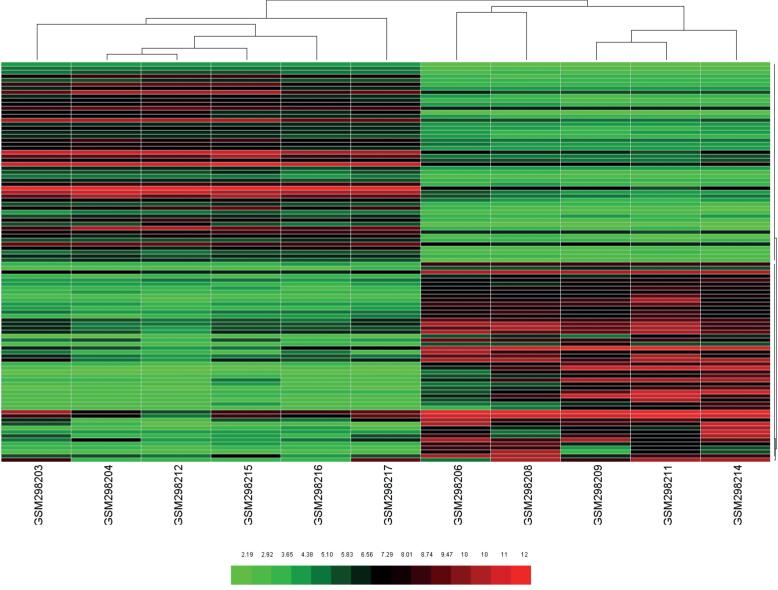
The heatmap of top fifty up-regulated genes and top fifty down-regulated genes.

Comparison between the ulcerative and the non-ulcerative IC groups yielded results indicating that there were hardly any DEGs between the two groups, we therefore chose to show the comparison results between ulcerative IC tissues and normal tissues. In total, 483 DEGs were identified in the gene expression profile GSE11783, including 216 up-regulated and 267 down-regulated genes. A volcano plot of all DEGs (Figure-1), as well as a heatmap (Figure-2) of the top 50 up-regulated and top 50 down-regulated genes, are presented.

### Enrichment analysis

**Figure 3:**
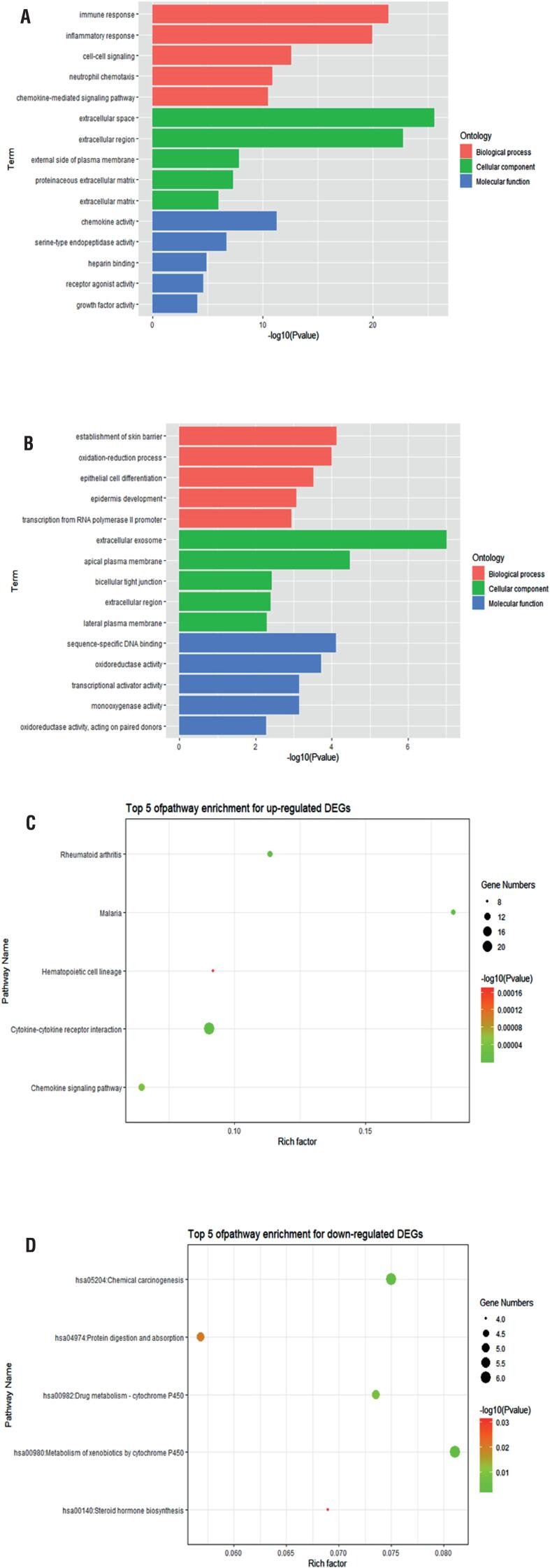
(a) The first five Gene Ontology (GO) enrichment analyses for up-regulated DEGs. (b) The first five GO enrichment analyses for down-regulated DEGs. The Kyoto Encyclopedia of Genes and Genomes (KEGG) pathway analysis of up-regulated (c) and down-regulated genes (d).

A GO functional enrichment analysis of the DEGs was performed using the DAVID online tool. The first five enrichment analyses for up-regulated DEGs (Figure-3a) and down-regulated DEGs (Figure-3b) are shown. In terms of BP analysis, the up-regulated DEGs were mainly enriched in immune response, inflammatory response, and cell-cell signaling. The down-regulated DEGs were mainly enriched in the establishment of the skin barrier, the oxidation-reduction process, and epithelial cell differentiation. In terms of CC analysis, the up-regulated DEGs were mainly enriched in the extracellular region, extracellular space, and external side of the plasma membrane. The downregulated DEGs were mainly enriched in the extracellular exosome, apical plasma membrane, and bicellular tight junction. In terms of MF analysis, the up-regulated DEGs were mainly enriched in chemokine activity, serine-type endopeptidase activity, and heparin binding. The down-regulated DEGs were mainly enriched in sequence-specific DNA binding, oxidoreductase activity, and transcriptional activator activity. Among them, the up-regulated and down-regulated DEGs were all mainly enriched in CC.A KEGG pathway enrichment analysis showed that the up-regulated DEGs were significantly enriched in cytokine-cytokine receptor interaction, malaria, and rheumatoid arthritis, while the down-regulated DEGs were mainly enriched in the metabolism of xenobiotics by cytochrome P450 (Figures 3c and 3d).

### Construction of the PPI network and determination of key modules

**Figure 4:**
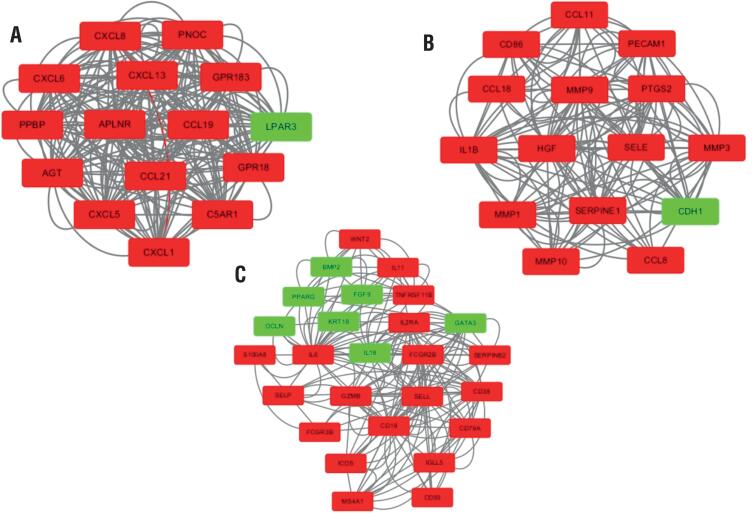
The key modules of PPI network. Module1(a), Module 2(b), Module3(c).

The analysis of DEGs using the STRING online tool resulted in the construction of a PPI network with a total of 461 nodes and 1618 edges. This PPI network was imported into the Cytoscape software. Subsequently, the MCODE plugin was used to select key modules, cluster1, cluster 2 and cluster 3 (Figure-4). Red represents up-regulated genes and green represents down-regulated genes. Only the genes in the first of these three modules had MCODE scores ≥12 (10). The DEGs in module 1 were selected for GO and KEGG analyses. Module 1 was significantly enriched in BP in the GO analysis. In the KEGG analysis, module 1 was mainly enriched in the chemokine signaling pathway and cytokine-cytokine receptor interactions (Table-1).

**Table 1:** The top 5 Enriched Gene Ontology (GO) and Kyoto Encyclopedia of Genes and Genomes (KEGG) of module 1

ID	Term	Count	P-Value	Genes
GO:0008009	chemokine activity	8	3.74E-15	CXCL6, CXCL8, CCL21, CXCL1, PPBP, CCL19, CXCL13, CXCL5
GO:0070098	chemokine-mediated signaling pathway	8	5.98E-14	CXCL6, CXCL8, CCL21, CXCL1, PPBP, CCL19, CXCL13, CXCL5
GO:0007186	G-protein coupled receptor signaling pathway	12	3.06E-12	CXCL6, CXCL8, CCL21, GPR183, APLNR, CXCL1, PPBP, CCL19, CXCL13, CXCL5, AGT, GPR18
GO:0006955	immune response	10	6.44E-12	CXCL6, CXCL8, CCL21, GPR183, C5AR1, CXCL1, PPBP, CCL19, CXCL13, CXCL5
GO:0006954	inflammatory response	9	1.67E-10	CXCL6, CXCL8, CCL21, C5AR1, CXCL1, PPBP, CCL19, CXCL13, CXCL5
hsa04062	Chemokine signaling pathway	8	2.85E-09	CXCL6, CXCL8, CCL21, CXCL1, PPBP, CCL19, CXCL13, CXCL5
hsa04060	Cytokine-cytokine receptor interaction	8	1.84E-08	CXCL6, CXCL8, CCL21, CXCL1, PPBP, CCL19, CXCL13, CXCL5

### Tissue-specific expression analysis of key modules

The genes of three key modules were analyzed using the BioGPS online tool. A total of 19 genes were identified. The primary tissue-specific expression system was the skin/muscular system (9/19, 47.4%), in which the genes were specifically expressed in smooth muscle tissue. The secondary tissue-specific expression system was the hematologic/immune system (5/19, 26.3%) (Table-2).

**Table 2:** The Tissue-specific expressed genes identified by BioGPS.

System	Genes
Hematologic/immune	CCL21, GPR18, C5AR1, PPBP, ICOS
Neurologic	SELE
Skin/ muscle	CXCL6, CXCL8, CXCL1, CXCL5, CCL11, MMP3, IL6, CCL8, MMP1
Reproductive	MMP10, GATA3
Endocrine	PPARG, TNFRSF11B

### Identification of hub gene

**Figure 5:**
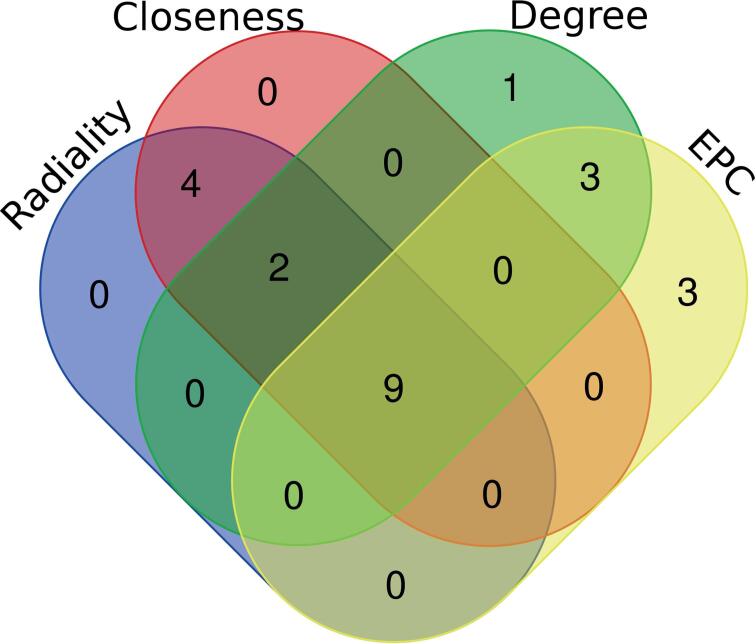
Nine Hub genes were identified by four methods of CytoHubba.

According to the four ranking methods in cytoHubba, the top 15 Hub genes identified by each ranking method are shown in Table-3. The intersection was obtained from the genes determined by these four methods, yielding nine Hub genes (Figure-5), including SELL, CXCL8, PECAM1, CD86, MMP9, CXCL1, IL6, PTGS2, and IL1B.

**Table 3:** The top 15 hub genes resulting from each ranking method.

Closeness	Degree	EPC	Radiality
PECAM1	PECAM1	PECAM1	PECAM1
PTGS2	PTGS2	MMP9	PTGS2
MMP9	MMP9	PTGS2	MMP9
BMP2	CXCL1	CXCL1	BMP2
THY1	CXCL8	CXCL13	THY1
PPARG	CD86	CCL19	PPARG
CXCL1	CXCL5	CD86	CXCL1
CXCL8	IL6	CXCL8	CXCL8
CD86	PPBP	CXCL5	CD86
IL6	HGF	CCL21	IL6
HGF	SELL	IL6	HGF
SELL	IL1B	PPBP	SELL
EPCAM	CD19	SELL	EPCAM
IL1B	CDH1	IL1B	IL1B
CDH1	IL18	IL18	CDH1

### Identification of possible core genes

Nine hub genes were identified by the PPI network. Tissue-specific expression analysis showed that three were specifically expressed in the skin/muscular system, and these three genes (CXCL8, CXCL1, and IL6) were considered to be possible core genes.

## DISCUSSION

IC/BPS is a chronic inflammatory disease of the bladder with unknown pathogenesis. However, several theories have been proposed regarding the cause of this syndrome, such as deficiency of glycosaminoglycans (GAGs) (12), increased urothelial permeability (13), immune response (14), and inflammatory response (15). In this study, a total of 483 DEGs were identified between ulcerative IC/BPS and controls, including 216 up-regulated and 267 down-regulated DEGs. The GO analysis showed that up-regulated and down-regulated DEGs were all mainly enriched in CC. The up-regulated DEGs were mainly enriched in the extracellular region, extracellular space, and external side of the plasma membrane, while the down-regulated DEGs were mainly enriched in the extracellular exosome, apical plasma membrane, and bicellular tight junction. Some studies have suggested that the long noncoding RNA MEG3 in urine exosomes can be used as a biomarker for IC/BPS (16). Stellavato et al. (17) suggested that continuous loss of extracellular matrix may be related to inflammation in IC/BPS. The KEGG pathway enrichment analysis indicated that DEGs were significantly enriched in the interaction between cytokines and cytokine receptors. Some studies have suggested that urinary cytokines can be used as biomarkers for IC/BPS (18).

The PPI network of DEGs was constructed using the STING online website and visualized with Cytoscape software. Three key modules were then identified using the MCODE plug-in. Only the genes in module 1 had MCODE scores greater than 12, so module 1 was considered the most important module. GO and KEGG analyses were performed on module 1, and the GO analysis indicated that module 1 was significantly enriched in chemokine activity, the chemokine-mediated signaling pathway, the G-protein coupled receptor signaling pathway, the immune response, and the inflammatory response. Tyagi et al. (5) suggested that chemokines are increased in the urine of patients with IC/BPS. G-protein coupled receptors can respond to a variety of input signals, thereby regulating various biological processes, so they are an important drug target (19). Some studies suggest that the immune response plays an important role in the pathophysiology of IC/BPS (20). Grover et al. (15) suggest that the inflammatory response plays a key role in the pathogenesis of IC/BPS. The KEGG analysis indicated that module 1 was mainly enriched in the Chemokine signaling pathway and Cytokine-cytokine receptor interaction. This is consistent with the results of previous KEGG analyses of DEGs.

BioGPS was used to perform an online analysis of DEGs in three key modules, and the results showed that DEGs were specifically expressed in the skin/muscular system, specifically in smooth muscle tissue, and the hematologic/immune system. Smooth muscle is widely distributed in the bladder, which may indirectly explain the clinical symptoms of IC/BPS that manifest in bladder tissue.

A PPI network of DEGs was also constructed to identify nine hub genes (SELL, CXCL8, PECAM1, CD86, MMP9, CXCL1, IL6, PTGS2, and IL1B) using four sequencing methods in cytoHubba, which were then intersected with the results of the tissue-specific expression analysis to obtain three possible core genes (CXCL8, CXCL1, and IL6). CXCL8, also known as IL-8, is a member of the chemokine family with proinflammatory effects (21). Some studies have found increasing levels of IL-8 in the urine of patients with IC/BPS, which is positively correlated with bladder mast cell counts (22, 23). However, some studies have suggested that IL-8 is a normal epithelial growth factor, and its expression level is reduced in patients with IC/BPS (24, 25). CXCL1, another member of the chemokine family, promotes central sensitization and nociceptors (26). Peters et al. (27) found that urinary CXCL1 levels were positively correlated with pain scores, urgency, and Interstitial Cystitis Symptom Problem Index scores in patients with IC/BPS. Furuta et al. (28) found that CXCL1 expression was significantly increased in the urine of IC/BPS patients compared to normal controls. IL6 is a multifunctional cytokine and considered to be a powerful proinflammatory factor (29, 30). Lai et al. (31) concluded that factor IL6 levels are higher in ulcerative interstitial cystitis than in non-ulcerative IC/BPS. These three core genes and their signaling pathways require further investigation to determine their relationship with IC/BPS. However, our study has several limitations. Further studies are therefore required to validate our results.

## CONCLUSIONS

In this study, 483 DEGs were identified, including three key modules and 9 and hub genes. Three possible core genes (CXCL8, CXCL1, IL6) were then found. These may be potential biomarkers of interstitial cystitis and have guiding significance for clinical treatment.
